# Panoramic Landmarks: Comparing LLM-Assisted, Manual Tracing, and Self-Directed Learning in Dental Education

**DOI:** 10.1016/j.identj.2025.109393

**Published:** 2026-01-22

**Authors:** Suresh Kandagal Veerabhadrappa, Jayanth Kumar Vadivel, Seema Yadav Roodmal, Thantrira Porntaveetus, Anand Marya, Siddharthan Selvaraj

**Affiliations:** a Department of Oral Diagnostic Sciences, Faculty of Dentistry, SEGi University, Petaling Jaya, Selangor, Malaysia; b Department of Oral Medicine and Radiology, Saveetha Dental College and Hospitals, Saveetha Institute of Medical and Technical Sciences, Chennai, India; c Department of Periodontics and Implantology, Faculty of Dentistry, SEGi University, Petaling Jaya, Selangor, Malaysia; d Center of Excellence in Precision Medicine and Digital Health, Chulalongkorn University Implant and Esthetic Center, Faculty of Dentistry, Chulalongkorn University, Bangkok, Thailand; e Clinic of General, Special Care and Geriatric Dentistry, Center for Dental Medicine, University of Zurich, Zurich, Switzerland; f Faculty of Dentistry, University of Puthisastra, Phnom Penh, Cambodia; g Department of Dental Research Cell, Dr. D. Y. Patil Dental College & Hospital, Dr. D. Y. Patil Vidyapeeth, Pune, India

**Keywords:** Artificial intelligence, Dental, Education sustainability, Landmarks, Panoramic radiography, Tracing

## Abstract

**Background:**

Accurate identification of anatomical landmarks on panoramic radiographs is a foundational yet challenging skill in dentistry. Traditional didactic teaching often requires supplementation to achieve proficiency. This study evaluates and compares the efficacy of three supplementary learning modalities: self-directed learning (SDL), traditional manual tracing (MT), and an AI-driven approach using ChatGPT.

**Methods:**

In this prospective study, 63 third-year dental students were assigned to one of three groups (*n* = 21 each): SDL, MT, or ChatGPT-assisted learning. Following a theoretical lecture, students were assessed using a 30-item test immediately after the lecture (baseline) and again at a 4-week follow-up. Intra- and intergroup differences were analysed using Wilcoxon signed-rank and Kruskal–Wallis tests, respectively.

**Results:**

Intergroup analysis demonstrated that the MT group achieved significantly higher overall scores than both the SDL and ChatGPT groups (*P* < .05), correctly identifying the most landmarks (26/30). Within-group analysis revealed significant improvements from baseline in the MT group for 24 landmarks (*P* < .05 for key structures like the hard palate and hyoid bone) and in the ChatGPT group for 16 landmarks (*P* < .05 for the glossopharyngeal air space). The SDL group showed no significant improvement. Notably, the ChatGPT group outperformed MT in identifying four specific landmarks, including the zygomatic process and nasopharyngeal air space.

**Conclusion:**

For optimal learning in dental radiology, an integrated approach is recommended. MT proved most effective overall, while ChatGPT added value for specific landmarks. Combining both methods may further enhance student proficiency.

**Clinical Relevance:**

Identification of landmarks is essential for accurate diagnosis and treatment planning. This study demonstrates that MT significantly enhances landmark recognition, while ChatGPT provides supplementary value. Integrating traditional and AI-assisted methods may further strengthen dental radiology education.

## Introduction

Dental panoramic radiograph (PAN) is a commonly used extraoral 2D imaging modality in dentistry. It provides a comprehensive panoramic view of the maxillary and mandibular dentition, along with their supporting structures.^1^ Due to its wide diagnostic applications, PAN is employed across various dental disciplines for screening and treatment planning.^2^ Recognizing its importance, dental curricula worldwide include mandatory instruction on the basic principles and interpretation of PAN for undergraduate dental students.

The interpretation of PAN depends on an accurate understanding and identification of anatomical structures. However, recognizing these landmarks can be challenging for learning students due to the complexity of the projection technique and the presence of multiple superimpositions and image distortions, often exacerbated by technical errors during image acquisition.^1^^,^^2^ Additionally, ghost images, artefacts, and the blurring or magnification of structures located outside the focal trough can further obscure anatomical landmarks, making their identification particularly difficult for students who are still developing familiarity with normal anatomical landmarks in PAN.^3^^,^^4^

Traditionally, the interpretation of PAN is taught through didactic lectures, textbook-based learning, and practical demonstrations of anatomical landmarks. Students also commonly engage in tracing exercises to reinforce their understanding of anatomical structures. However, traditional methods often lack the depth of perception needed to fully grasp the anatomical features.^5^ This limitation can make it difficult for students to accurately identify, recognize, and memorize the landmarks. Furthermore, the current generation of learners tends to prefer alternative, technology-enhanced teaching approaches over conventional methods.^6^ In response to this shift, researchers have developed virtual reality (VRTs) techniques to teach radiographic anatomy. While students have reported positive feedback regarding their VRTs learning experience but no significant improvement in their ability to identify individual landmarks in PANs was reported. Moreover, VRTs may not be feasible for all educational institutions, particularly those with limited financial resources, due to the high cost of implementing such systems.^4^

The emergence of ChatGPT, a conversational large language model developed by OpenAI, has generated growing interest in its application within university-based dental education. Since its public release at the end of 2022, ChatGPT has gained popularity for its use in content explanation, learner support, interactive learning, patient communication, clinical simulation, and personalized tutoring.^7^^,^^8^ It is designed to generate human-like responses by interpreting natural language prompts and drawing from a vast corpus of textual data, enabling its application across diverse domains, including healthcare and education.^9^ Over recent years, ChatGPT has advanced through successive models such as GPT-3.5 and GPT-4, each offering progressively enhanced reasoning, accuracy, and language generation capabilities.^10^^,^^11^

A recent systematic review on the application of ChatGPT in dental education highlights its potential as a promising supportive tool. However, its integration into dental curricula remains largely informal and supplementary, with limited structured implementation.^8^ Previous studies have shown that ChatGPT-3 is effective in explaining pathological conditions, radiographic features, and anatomical landmarks in PANs.^7^ Moreover, ChatGPT offers a cost-effective and easily accessible alternative to traditional teaching methods, particularly in institutions with resource limitations or faculty shortages.^7^^,^^9^

Despite these advantages, there is a paucity of literature evaluating the effectiveness of ChatGPT in enhancing undergraduate dental students’ understanding and identification of anatomical landmarks on PAN.^12^ Therefore, this study aims to assess the effectiveness of AI-supported learning using ChatGPT as a supplemental tool for improving students’ comprehension and identification of anatomical landmarks on PANs. In addition, students’ understanding was compared with other instructional strategies, including self-directed learning (SDL) and manual tracing (MT).

## Methods

### Study design, setting, and duration

This comparative study was conducted among Year 3 Bachelor of Dental Surgery (BDS) students in the Department of Oral Diagnostic Sciences, Faculty of Dentistry, SEGi University, Malaysia during the radiology module over a 4-week period from March to April 2025.

### Inclusion and exclusion criteria

Dental students who have attended introductory lecture on PAN and willingness to participate and complete all components of the study were included. Prior exposure to AI-assisted radiographic tools and incomplete participation in any phase of the study were excluded.

### Study participants and groupings

At the beginning of the study, all participants attended a compulsory 1-hour theoretical lecture delivered by a board-certified oral radiologist. The session covered the fundamental concepts and radiographic appearances of anatomical landmarks on PAN. Immediately after the lecture, students completed a 30-item written assessment to determine their baseline knowledge. In this assessment, they were required to identify each landmark within 30 minutes on a standard PAN. Following the evaluation of baseline scores, the students were randomly allocated into three equal groups (*n* = 21 each) using a computer-generated randomization sequence (Figure 1).

Group A: SDL: Students used conventional learning resources, including textbooks, lecture notes, and instructional videos, to independently study anatomical landmarks on PAN.
Group B: MT: Students traced anatomical landmarks on a standard PAN using tracing paper. A checklist of key landmarks was provided, and students were instructed to trace and label each structure accordingly.
Group C: ChatGPT-assisted learning: Students engaged with an AI-powered chatbot (ChatGPT 4.0) to interactively study PAN landmarks. Prior to the session, they were trained on effective usage strategies, including conversational queries, use of interactive prompts, and leveraging personalized feedback. Students were instructed to ask ChatGPT specific prompts regarding each landmark’s name, radiographic features, and location. At the end of the session, participants submitted compiled summary notes describing the radiographic appearance and anatomical position of each landmark.

**Fig:**
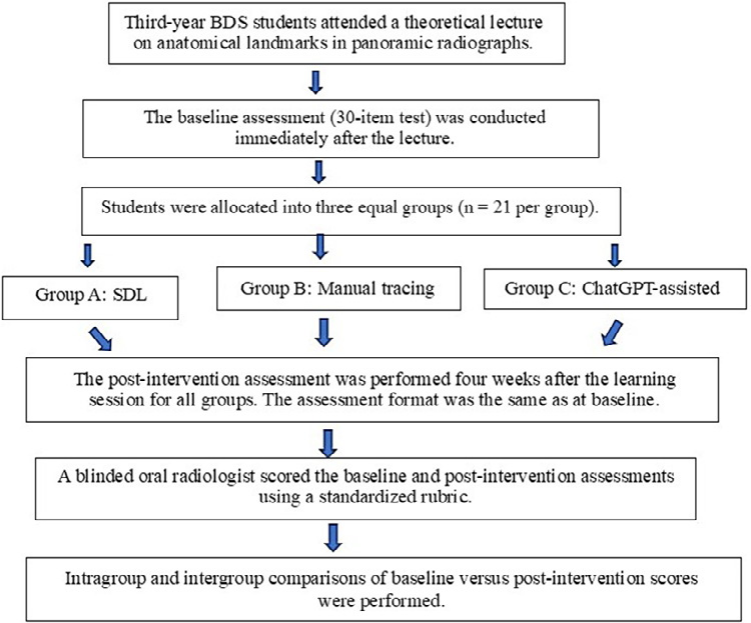
Flowchart of the study.

### Post intervention assessment

Four weeks after the completion of the learning sessions, a postintervention assessment was administered to evaluate performance and knowledge retention. This assessment consisted of a 30-item written test using standardized PANs, in which students were required to identify anatomical landmarks within a 30-minute limit.

Scoring was performed by a single board-certified oral and maxillofacial radiologist with over 10 years of experience. A standardized rubric was applied, awarding 1 mark for each correct response and 0 for incorrect or missing answers. To ensure reliability, the assessor underwent a prestudy calibration using 25 PANs. A consensus ‘gold standard’ for these images was established by two independent senior radiologists, and the study assessor achieved >90% agreement with this standard. The assessor was completely blinded to group allocation and was not involved in the intervention procedures. This single-assessor approach was utilized to eliminate interexaminer variability while ensuring high diagnostic accuracy

### Ethical consideration and confidentiality

An ethical approval was obtained from the Institutional Research Board (SEGiEC/SR/FOD/13/2024-2025). All students were informed about the study’s objectives, methodology, and confidentiality measures. The study commenced only after obtaining written informed consent. No personally identifiable information was collected from the students.

### Statistical analysis

Data were analysed using IBM SPSS Statistics software (Version 26; IBM Corp.). Intragroup comparisons (baseline vs postintervention) were performed using the Wilcoxon signed-rank test, while intergroup comparisons of student performance were evaluated using the Kruskal–Wallis test. A *P* value of <.05 was considered indicative of statistical significance.

## Results

All participants were third-year BDS students aged 21 to 23 years, predominantly female, and of Malaysian nationality.

### Intragroup comparison of baseline and postinstructional mean scores (Table 1)

Students in Group 1 (SDL) showed postinstructional improvements in mean scores for 8 out of 30 landmarks which include pterygomaxillary fissure, floor of the orbit, inferior concha, hard palate, coronoid process, inferior border of the mandible, sigmoid notch, and glossopharyngeal air space. However, none of these changes were statistically significant. In contrast, postinstructional scores for most of the remaining landmarks decreased compared to baseline. A statistically significant decline was observed for the cervical vertebra (*P* < .01) (Table 1).

**Table 1:** Intragroup comparison of baseline and postinstructional mean scores for the identification of anatomical landmarks across the three groups.

Category	Landmarks	Group 1: SDL		*P* value	Group 2: Manual tracing		*P* value	Group 3: ChatGPT		*P* value
		Baseline mean score	Postinstructional mean score		Baseline mean score	Postinstructional mean score		Baseline mean score	Postinstructional mean score	
Maxillary	1. Articular tubercle	0.86	0.76	.68	0.71	1.00	.25	0.75	0.79	1.00
	2. Zygoma	0.81	0.67	.50	0.71	0.86	1.00	0.75	0.75	1.00
	3. Zygomatic process of maxilla	0.71	0.57	.50	0.76	0.71	1.00	0.71	0.75	1.00
	4. Pterygomaxillary fissure	0.57	0.67	.77	0.71	0.76	.38	0.62	0.67	1.00
	5. Floor of orbit	0.71	0.81	.72	0.86	0.9	.28	0.75	0.83	.75
	6. Inferior concha	0.62	0.76	.50	0.48	0.86	.22	0.54	0.67	.50
	7. Nasal septum	0.81	0.48	.09	0.86	0.9	.68	0.79	0.67	.50
	8. Anterior nasal spine	0.9	0.67	.17	0.71	1.00	.5	0.83	0.83	1.00
	9. Floor of maxillary sinus	0.76	0.57	.38	0.90	0.71	1.00	0.79	0.54	.14
	10. Hard palate	0.48	0.57	.75	0.52	0.90	<.01*	0.54	0.58	1.00
Mandible	11. Condyle	0.95	0.90	1.00	1.0	1.00	1.00	0.96	0.96	.62
	12. Neck of the condyle	0.95	0.86	.62	1.0	1.00	1.00	0.96	0.96	.62
	13. Coronoid process	0.81	0.86	1.00	0.81	0.95	.37	0.83	0.83	1.00
	14. Inferior alveolar canal	0.81	0.67	.50	0.81	0.86	1.00	0.83	0.83	1.00
	15. Inferior border of mandible	0.71	0.81	.68	0.76	0.95	.12	0.75	0.83	.75
	16. Mental foramen	0.86	0.71	.50	0.86	0.90	1.00	0.88	0.71	.34
	17. Submandibular fossa	0.52	0.38	.50	0.76	0.48	1.00	0.58	0.59	.42
	18. Mandibular angle	0.86	0.71	.37	0.90	0.95	.62	0.83	0.79	1.00
	19. External oblique ridge	1.19	0.57	.50	0.76	0.90	.21	1.17	0.79	1.00
	20. Sigmoid notch	0.52	0.62	.72	0.71	0.57	1.00	0.54	0.58	1.00
Mid line structures	21. Hyoid bone	0.57	0.38	.28	0.52	0.95	<.02*	0.58	0.71	.58
	22. Cervical vertebra	0.67	0.24	<.01*	0.76	0.76	.75	0.67	0.71	1.00
Ghost image	23. Ghost image of mandible	0.33	0.24	.75	0.43	0.86	<.001*	0.33	0.62	.11
Soft tissues shadows	24. Ear lobe	0.86	0.67	.34	0.86	0.81	1.00	0.83	0.62	.22
	25. Soft palate and uvula	0.67	0.43	.22	0.81	0.86	.28	0.67	0.62	1.00
	26. Tongue	0.86	0.52	.06	0.86	0.81	1.00	0.83	0.67	.34
	27. Lip line	0.29	0.29	1.00	0.52	0.67	.05	0.38	0.46	.80
Air spaces	28. Palatoglossal air space	0.43	0.38	1.00	0.67	0.81	<.03*	0.42	0.62	.26
	29. Naso pharyngeal air space	0.43	0.38	1.00	0.38	0.52	.72	0.46	0.58	.58
	30. Glossopharyngeal air space	0.19	0.43	.22	0.48	0.90	<.001*	0.21	0.62	<.02*

The asterisk (*) indicates statistically significant results.

Students in Group 2 (MT) demonstrated improvements in mean scores for 24 out of 30 landmarks. Statistically significant gains were observed for the hard palate (*P* < .01), hyoid bone (*P* < .02), ghost image of the mandible (*P* < .001), palatoglossal air space (*P* < .03), and glossopharyngeal air space (*P* < .001). Slight reductions in postinstructional mean scores were noted for the floor of the maxillary sinus, submandibular fossa, external oblique ridge, ear lobe, and tongue, while no change in the mean score was observed for the zygomatic process of the maxilla (Table 1).

Students in Group 3 (ChatGPT-assisted learning) showed improvements in mean scores for 16 out of 30 landmarks, with a statistically significant gain for the glossopharyngeal air space (*P* < .02). No changes in the mean scores were observed for the zygomatic process of the maxilla, anterior nasal spine, condyle, neck of the condyle, coronoid process, and inferior alveolar canal. Conversely, reductions in mean scores were observed for the nasal septum, floor of the maxillary sinus, mental foramen, mandibular angle, external oblique ridge, ear lobe, soft palate and uvula, and tongue, though these differences were not statistically significant.

### Intergroup comparison of postinstructional mean scores (Table 2)

The postinstructional mean scores were higher for Group 2 in 26 out of 30 anatomical landmarks, with statistically significant gains observed for the nasal septum (*P* < .02), anterior nasal spine (*P* < .03), external oblique ridge (*P* < .04), hyoid bone (*P* < .001), cervical vertebra (*P* < .001), ghost image of the mandible (*P* < .001), soft palate and uvula (*P* < .02), palatoglossal air space (*P* < .02), and glossopharyngeal air space (*P* < .01). Students in Group 3 demonstrated higher mean scores compared to the other two groups for the zygomatic process of the maxilla, submandibular fossa, and nasopharyngeal air space; however, these differences were not statistically significant (Table 2).

**Table 2:** Intergroup comparison of postinstructional mean scores for the identification of anatomical landmarks across the three groups.

Category	Landmark	Group 1: SDL mean postinstructional score	Group 2: Manual tracing mean postinstructional score	Group 3: ChatGPT mean postinstructional score	*P* value
Maxillary	1. Articular tubercle	0.76	1.00	0.79	.21
	2. Zygoma	0.67	0.86	0.75	.35
	3. Zygomatic process of maxilla	0.57	0.71	0.75	.20
	4. Pterygomaxillary fissure	0.67	0.76	0.67	.78
	5. Floor of orbit	0.81	0.90	0.83	.56
	6. Inferior concha	0.76	0.86	0.67	.76
	7. Nasal septum	0.48	0.90	0.67	<.02*
	8. Anterior nasal spine	0.67	1.00	0.83	<.03*
	9. Floor of maxillary sinus	0.57	0.71	0.54	.67
	10. Hard palate	0.57	0.90	0.58	.09
Mandible	11. Condyle	0.90	1.00	0.96	.65
	12. Neck of the condyle	0.86	1.00	0.96	.40
	13. Coronoid process	0.86	0.95	0.83	.70
	14. Inferior alveolar canal	0.67	0.86	0.83	.14
	15. Inferior border of mandible	0.81	0.95	0.83	.46
	16. Mental foramen	0.71	0.90	0.71	.44
	17. Submandibular fossa	0.38	0.48	0.59	.79
	18. Mandibular angle	0.71	0.95	0.79	.21
	19. External oblique ridge	0.57	0.90	0.79	<.04*
	20. Sigmoid notch	0.62	0.57	0.58	.84
Mid line structures	21. Hyoid bone	0.38	0.95	0.71	<.001*
	22. Cervical vertebra	0.24	0.76	0.71	<.001*
Ghost image	23. Ghost image of mandible	0.24	0.86	0.62	<.001*
Soft Tissues shadows	24. Ear lobe	0.67	0.81	0.62	.65
	25. Soft palate and uvula	0.43	0.86	0.62	<.02*
	26. Tongue	0.52	0.81	0.67	.16
	27. Lip line	0.29	0.67	0.46	.06
Air spaces	28. Palatoglossal air space	0.38	0.81	0.62	<.02*
	29. Nasopharyngeal air space	0.38	0.52	0.58	.22
	30. Glossopharyngeal air space	0.43	0.90	0.62	.01*

The asterisk (*) indicates statistically significant results.

For Group 1, postinstructional mean scores were comparable to Group 3 for landmarks such as the pterygomaxillary fissure and mental foramen. Interestingly, the highest mean score for the sigmoid notch was observed in Group 1 compared to both Group 2 and Group 3. Slightly higher mean scores were also noted in Group 1, compared to Group 3, for the floor of the maxillary sinus, coronoid process, and ear lobe however they are not statistically significant (Table 2).

## Discussion

In this study, 30 anatomical landmarks including those of the maxilla, mandible, midline structures, ghost images, soft tissue shadows, and air spaces were assessed, as these are fundamental for dental students in accurately interpreting PANs and diagnosing pathologies. Incorrect identification of these landmarks may lead to diagnostic errors, ultimately affecting treatment planning and appropriate referrals.^13^ Previous studies have primarily examined students’ knowledge of PAN landmarks,^12^^,^^14^^,^^15^ yet the role of ChatGPT as supplementary instructional resources for learning these landmarks remains unexplored.

In our study, intragroup comparison within the SDL group showed only marginal postinstructional improvements in a few landmarks, while most declined relative to baseline score, reflecting the passive nature of SDL and lack of engagement that hinders memorization. In contrast, the MT group demonstrated consistent improvements, with significant gains for the hard palate, hyoid bone, lip line, palatoglossal air space, and glossopharyngeal air space, indicating that tactile and visual engagement enhances spatial recognition.^16^ Prior literature similarly supports tracing as an effective active-learning strategy.^16^^,^^17^ The ChatGPT group improved in 16 of 30 landmarks, with a significant gain for the glossopharyngeal air space; however, while ChatGPT offered interactive explanations and personalized feedback, overall improvements surpassed SDL but remained lower than MT.

Elsheikhi et al^12^ reported that fourth-year BDS students and interns reliably identified bony landmarks such as the condylar head, mandibular canal, and mental foramen, while nonbony structures like the middle nasal concha and meatus were more challenging. Their study, however, assessed only 24 landmarks, excluding key structures such as air spaces, ghost images, and soft tissue shadows. Similarly, Maeda et al^3^ found higher accuracy for bony landmarks than nonbony landmarks, including the contralateral nasopalatine line, cervical vertebrae, mandibular foramen, mastoid air cells, posterior pharyngeal wall, tongue, and nasal conchae and meatuses. In line with these findings, our study showed higher baseline scores for bony landmarks compared to nonbony ones immediately after the lecture, but postinstructional scores for both declined in the SDL group after 1 month. This suggests that initial recall relied on rote memorization, which diminished without reinforcement, indicating that SDL alone may be insufficient for long-term retention of radiographic anatomy.

Students in the MT group achieved higher postinstructional scores for complex landmarks such as the ghost image of the mandible, soft palate and uvula, lip line, palatoglossal arch, and nasopharyngeal and glossopharyngeal air spaces. This likely reflects the tracing process, which required active delineation of borders and contours, reinforcing their identification memory.^3^^,^^16^ ChatGPT outperformed SDL in several landmarks and even exceeded MT for the zygomatic process of the maxilla, submandibular fossa, and nasopharyngeal air space, though without statistical significance. These results suggest ChatGPT is a useful supplementary tool, but MT remains superior for landmark identification. Interestingly, the MT group demonstrated superior performance in identifying landmarks located outside the dentoalveolar region. This finding is clinically significant, as these structures are vital for the detection of systemic conditions and pathologies, including those associated with obstructive sleep apnoea (OSA). This suggests that the tactile and deliberate nature of MT may encourage a more comprehensive visual scan of the radiograph, preventing students from focusing solely on the dentition and ensuring they develop the diagnostic skills necessary to contribute to a patient’s overall systemic health.

Compared to MT, the ChatGPT group showed lower postinstructional scores for certain landmarks, partly due to its text-based explanations lacking visualization and tactile reinforcement essential for nonbony structures. While ChatGPT aided conceptual understanding, students with limited technological proficiency, poor prompt formulation, or difficulty critically evaluating AI content may have struggled to interact with the AI tool.^18^ Emerging literature supports its potential to enhance learning but stresses the need for structured guidance to maximize effectiveness.^7^^,^^8^^,^^19^, ^20^, ^21^

Previous research reveals that predominantly osseous structures are easier to recognize on PAN due to their well-defined borders, distinct radiographic outlines, and consistent anatomical positioning.^3^^,^^12^ Conversely, landmarks such as soft tissue shadows, air spaces, and ghost images are more challenging to identify due to indistinct margins, low contrast, and frequent superimposition on adjacent structures. Moreover, ghost images in PAN are often distorted and duplicated, further complicating interpretation, particularly for learners with limited familiarity with PAN projection geometry.^3^^,^ 13, 14, 15

The literature on generative AI shows promising performance in dental education and assessment.^22^^,^^23^ Previous studies demonstrate that these models can perform well on dental licensing examination multiple-choice questions in the United States and the United Kingdom, with ChatGPT-4.0 consistently outperforming ChatGPT-3.5 in accuracy and reasoning.^22^^,^^23^ Its strongest results were observed in prosthodontic and restorative dentistry questions from the National Board Dental Examination and the UK Overseas Registration Examination.^23^ In paediatric dentistry, AI tools remain valuable as educational adjuncts for providing general patient information.^24^ Generative AI has also shown potential in research support, as chatbots can streamline components of systematic reviews such as screening and data extraction.^25^ Additionally, ChatGPT has demonstrated superior responsiveness and quality when addressing oral cancer–related questions, highlighting its utility in patient education and clinical communication.^26^

Our results indicate that ChatGPT-4.0 assisted learning supports the comprehension of panoramic anatomical knowledge but does not outperform MT for most landmarks. Unlike licensing examination studies, which assess text-based clinical decision-making, our study focuses on the spatial interpretation of radiographic landmarks a domain in which AI-generated textual explanations may be insufficient without visual or tactile reinforcement. Nonetheless, our finding that ChatGPT outperformed SDL for several landmarks was consistent with current literature, supporting its role as an accessible supplementary tool that can enhance understanding when used with structured guidance.

The findings of this study highlight the importance of integrating active and interactive learning strategies into dental radiology for learning anatomical landmarks. MT enhances spatial understanding through tactile reinforcement, while AI-assisted platforms such as ChatGPT offer interactive, self-paced learning with individualized explanations and round-the-clock accessibility, particularly beneficial for revision.^27^^,^^28^ A blended approach combining tracing with AI-based tools may optimize learning outcomes.

The present study has certain limitations. The sample size was constrained by the fixed enrolment of the third-year class (*n* = 21 per group). While nonparametric tests were employed to account for this, the relatively small sample, combined with the single-centre design and a 1-month follow-up period, may limit the generalizability of the findings to other academic levels and may not adequately capture long-term retention of anatomical landmark knowledge. The reliance on postinstructional mean scores as the primary outcome measure may also not fully reflect students’ clinical application skills or diagnostic accuracy. Moreover, the effectiveness of ChatGPT is inherently dependent on the quality of prompts and students’ ability to interact effectively with the AI, which may have influenced the outcomes. Furthermore, no formal correction for multiple comparisons was applied due to the exploratory nature of the study. Therefore, the findings regarding specific landmarks should be interpreted with caution. Future research should explore the integration of digital tracing, AI-assisted platforms, and structured SDL in multicentre studies to evaluate their long-term educational effectiveness, enhance external validity, and assess the reproducibility of results across diverse educational settings.

## Conclusion

The findings demonstrate that MT produced consistent and statistically significant improvements, particularly for air spaces, soft tissue shadows, and ghost images. ChatGPT-assisted learning outperformed SDL in several anatomical regions, highlighting its potential as a supplementary tool, though its overall effectiveness remained lower than MT. These results suggest that effective integration of AI platforms requires structured guidance, training in prompt formulation, and critical evaluation of AI-generated content.

## Author contributions

Conceptualization: Suresh Kandagal Veerabhadrappa, Seema Yadav Roodmal. Methodology: Suresh Kandagal Veerabhadrappa. Validation: Anand Marya, Jayanth Kumar Vadivel, Thantrira Porntaveetus. Formal analysis: Siddharthan Selvaraj, Suresh Kandagal Veerabhadrappa. Investigation: Suresh Kandagal Veerabhadrappa. Writing – original draft: Suresh Kandagal Veerabhadrappa. Writing – review and editing: Suresh Kandagal Veerabhadrappa, Seema Yadav Roodmal, Siddharthan Selvaraj. Visualization: Jayanth Kumar Vadivel, Anand Marya, Thantrira Porntaveetus. Supervision: Jayanth Kumar Vadivel, Anand Marya, Thantrira Porntaveetus.

## Declaration of generative AI and AI-assisted technologies in the writing process

During the preparation of this work the author used ChatGPT to check gramma of this manuscript. After using this tool/service, the author reviewed and edited the content as needed and takes full responsibility for the content of the publication.

## Data availability

The data that support the findings of this study are available from the corresponding author upon reasonable request.

## Conflict of interest

TP is currently serving as an editorial board member for the International Dental Journal but was not involved in the handling or review of this manuscript. All other authors declare no conflicts of interest.
